# Supplementary material for: Senolytic therapy combining Dasatinib and Quercetin restores the chondrogenic phenotype of human osteoarthritic chondrocytes by the release of pro‐anabolic mediators
Source: Aging Cell. 2024 Oct 14;24(1):e14361. doi: 10.1111/acel.14361 (PMC11995296; doi:10.1111/acel.14361)
Supplement: Supplementary file 1 — Appendix S1. [file ACEL-24-e14361-s001.pdf]

Supplementary

Materials and Methods:

S1: Materials and Methods

a) **Medium:**
   - Serum-free medium – human:
     DMEM 1 g/L glucose (Gibco Life Technologies), 1% Na-pyruvate (Merck Biochrom), 1% non-essential amino acids (NEAAs) (Merck Biochrom), 2 mM L-glutamine (PAN-Biotech), 0.5% penicillin/streptomycin (PAN-Biotech), 10 µg/mL 2-phospho-L-ascorbic acid trisodium salt (Sigma-Aldrich), 1% 100x insulin-transferrin-selenium (ITS) (Gibco, Life Technologies)
   - Serum-free medium – porcine:
     DMEM 4.5 g/L glucose (Gibco Life Technologies), 1% Na-pyruvate, 1% NEAAs, 2 mM L-glutamine, 1% penicillin/streptomycin, 10 µg/mL 2-phospho-L-ascorbic acid trisodium salt, 1% 100x ITS
   - Serum-containing medium – human:
     1:1 Ham's F12 (PAN-Biotech) and DMEM 1 g/L glucose, 10% heat inactivated fetal bovine serum (FBS) (PAN-Biotech), 0.5% penicillin/streptomycin, 2 mM L-glutamine, 10 µg/mL 2-phospho-L-ascorbic acid trisodium salt
   - Chondrogenic differentiation medium – human:
     DMEM 4.5 g/L glucose, 1% penicillin/streptomycin, 2 mM L-Glutamine, 0.1 µM Dexamethasone (Sigma-Aldrich), 0.2 mM 2-phospho-L-ascorbic acid trisodium salt, 40 µg/mL L-proline, 1 mM Na-pyruvate, 10 ng/mL of rhTGF-β3 (PeproTech) and 10 ng/mL rhBMP-6 (PeproTech), 1% 100x insulin-transferrin-selenium (ITS) (Gibco, Life Technologies)
   - Serum-containing medium – ATDC5 cells:
     1:1 DMEM 1 g/L glucose, HAM's F12, 5% FBS, 1% L-glutamine, 10 µg/mL Transferrin human (Sigma Aldrich), 0.03 µM Sodium Selenite (Sigma Aldrich), 1% penicillin/streptomycin
   - Chondrogenic differentiation medium – ATDC5 cells:
     DMEM 4.5 g/L glucose, 1% penicillin/streptomycin, 5% FBS, 2 mM L-Glutamine, 0.1 µM Dexamethasone (Sigma-Aldrich), 0.2 mM 2-phospho-L-ascorbic acid trisodium salt, 40 µg/mL L-proline, 1 mM Na-pyruvate, 10 ng/mL of rhTGF-β3 (PeproTech) and 10 ng/mL rhBMP-6 (PeproTech), 1% 100x insulin-transferrin-selenium (ITS) (Gibco, Life Technologies)

b) **Probes and Primers:**
   - TaqMan assays – human:
     Reference genes: GAPDH (Hs02758991_g1), HPRT1 (Hs02800695_m1), and TMEM199 (Hs01022209_m1)
     Target genes:
     COL2A1 (Hs00264051_m1), ACAN (Hs00153936_m1), CXCL1 (Hs00605382_gH), IL6 (Hs00174131_m1), MMP13 (Hs00233992), CDKN1A (Hs00355782_m1), CDKN2A (Hs00923894_m1), YAP1 (Hs00902712_g1) SOX9 (Hs00165814_m1), FGFR1 (Hs00241111_m1), FGFR3 (Hs00179829_m1)
   - Primers – human:

Target genes: IGF1 (forward 5’CTCTTCAGTTCGTGTGTGGAGAC; reverse 5’CAGCCTCCTTAGATCACAGCTC3’), FGF18 (all in one BioCat HQP088256-GC)

- TaqMan assays and primers – murine:
  Reference genes: GAPDH (Mm99999915_g1), HPRT1 (Mm03024075_m1)
  Target genes: ACAN (forward 5’AACTTCTTTGCCACCGGAGA3’; reverse 3’GGTGCCCTTTTTACACGTGAA5’), COL2A1 (forward 5’CCTGTCTGCTTCTTGTAAAAC3’; reverse 3’TGGGTATCATCAGGTCAGGT5’).

S2: Morphological Changes of hAC through Passaging

Morphological changes due to *in vitro* culturing are demonstrated by a correlation of cell length with increasing passage.

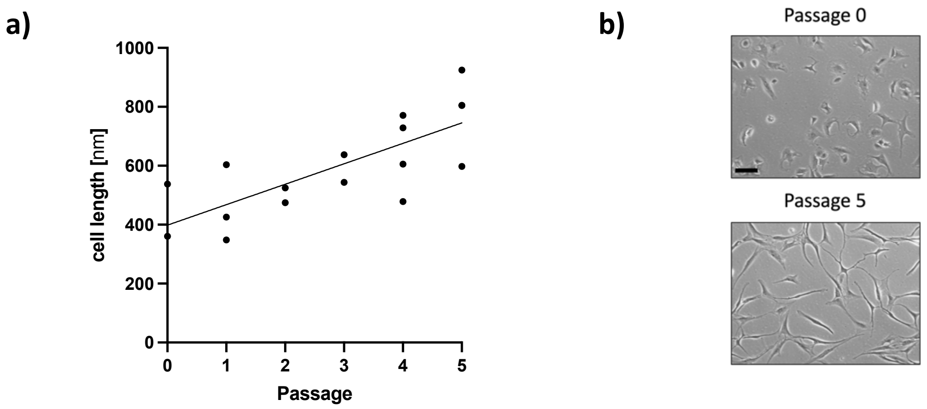

Figure S2: a) Passage dependent cell length (passage 0-5) (n≥2) and b) exemplary images of passage 0+5 (scale bar = 100 µm).

S3: Enhanced Chondroanabolism in Cartilage Tissue through D+Q Therapy

COL2 staining intensity of cartilage tissue was increased after senolytic treatment with D+Q at day 14.

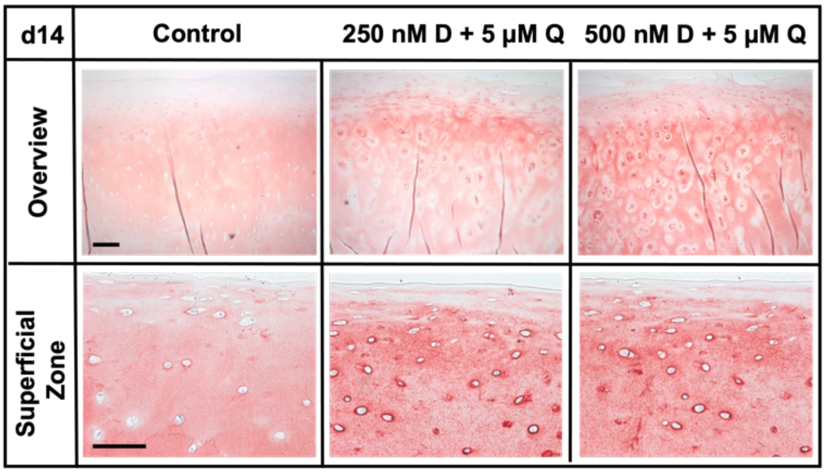

Figure S3: Exemplary COL2 staining (d14) of cartilage tissue w/o or with D+Q therapy (scale bar: overview = 200 µm, superficial zone = 100 µm).

S4: Navitoclax Therapy Reveals no Chondroanabolic Effects

Navitoclax therapy displays greater cytotoxic effects in high passage hAC (Figure S4a-b). Furthermore, p16-positive cells were reduced through therapy in cartilage tissue (Figure S4c). However, Navitoclax treatment reduced gene expression levels of COL2A1 and suppressed the chondrogenic redifferentiation of high passage hAC (Figure S4d-i).

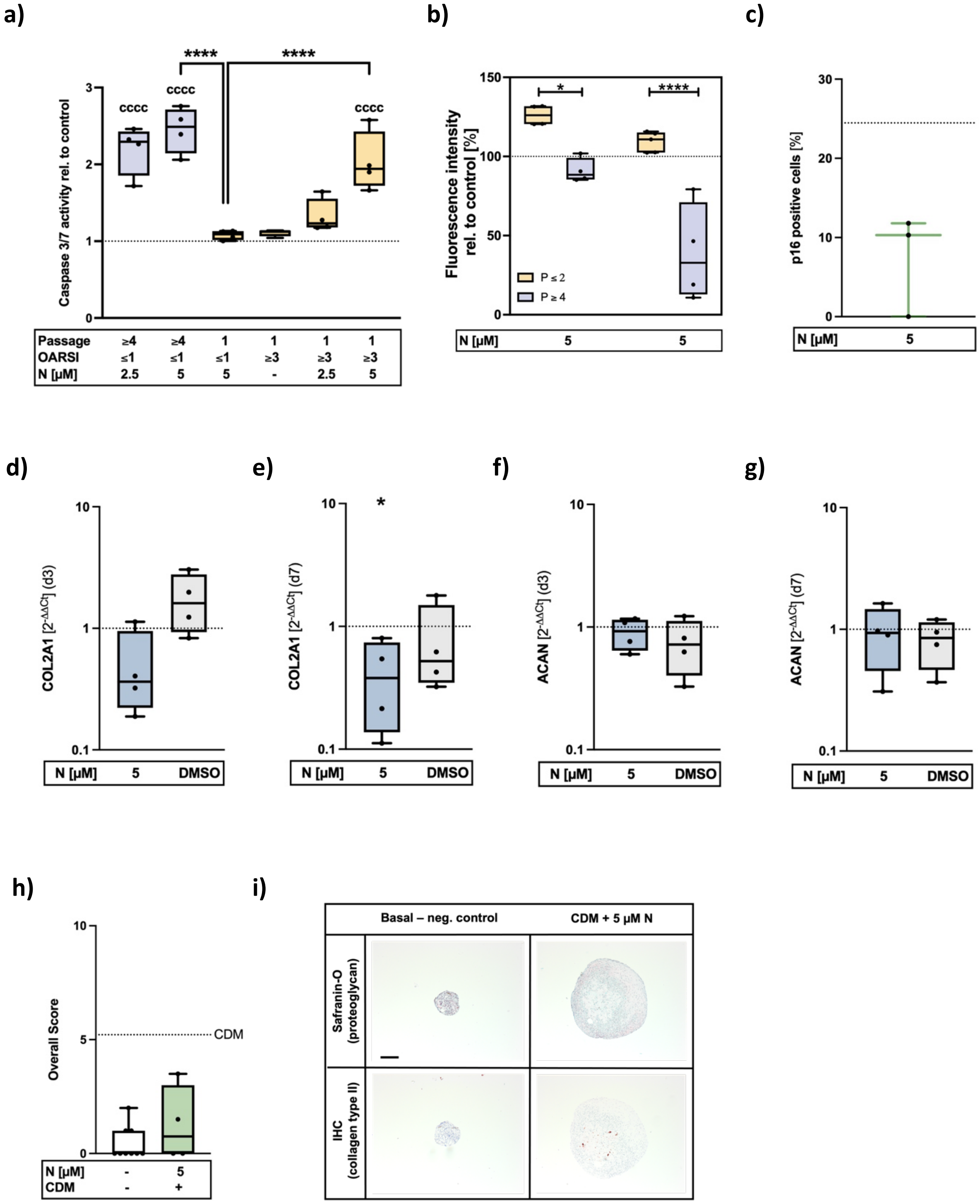

Figure S4: Senolytical therapy with Navitoclax. a) Caspase 3/7 activity of high vs. low passage hAC w/o or with Navi (2.5-5 mM) treatment (n=4). b) FI (alamarBlue assay) of low (P≤2) vs. high passage (P≥4) hAC after treatment with Navitoclax (n≥4). c) p16 positive cells in cartilage tissue (IHC staining) (n=3). d-e) Gene expression levels of COL2A1 (n=4) and f-g) ACAN (n=4) at d3+7 after Navitoclax therapy. h)

Overall score of the chondrogenic redifferentiation potential after Navitoclax therapy of high passage hAC (n≥4) and i) exemplary images of the safranin-O and COL2 staining (scale bar = 200 µm). Statistical analysis: one-way/ two-way ANOVA with Šidák's multiple comparisons test: a)-b) *p<0.05, ****p<0.0001; e) unpaired t-test: *p<0.05.

S5: Downregulation of SASP Factors on Gene Expression Level after Therapy with D+Q

Senolytic treatment resulted in a suppression of the gene expression levels CXCL1 and IL6 directly after therapy (d3; Figure S5a+c). After deprivation of D+Q the mRNA levels were no longer reduced (Figure S5b+d). The protease MMP13 gene expression level was attenuated at d3+7 (Figure S5e+f).

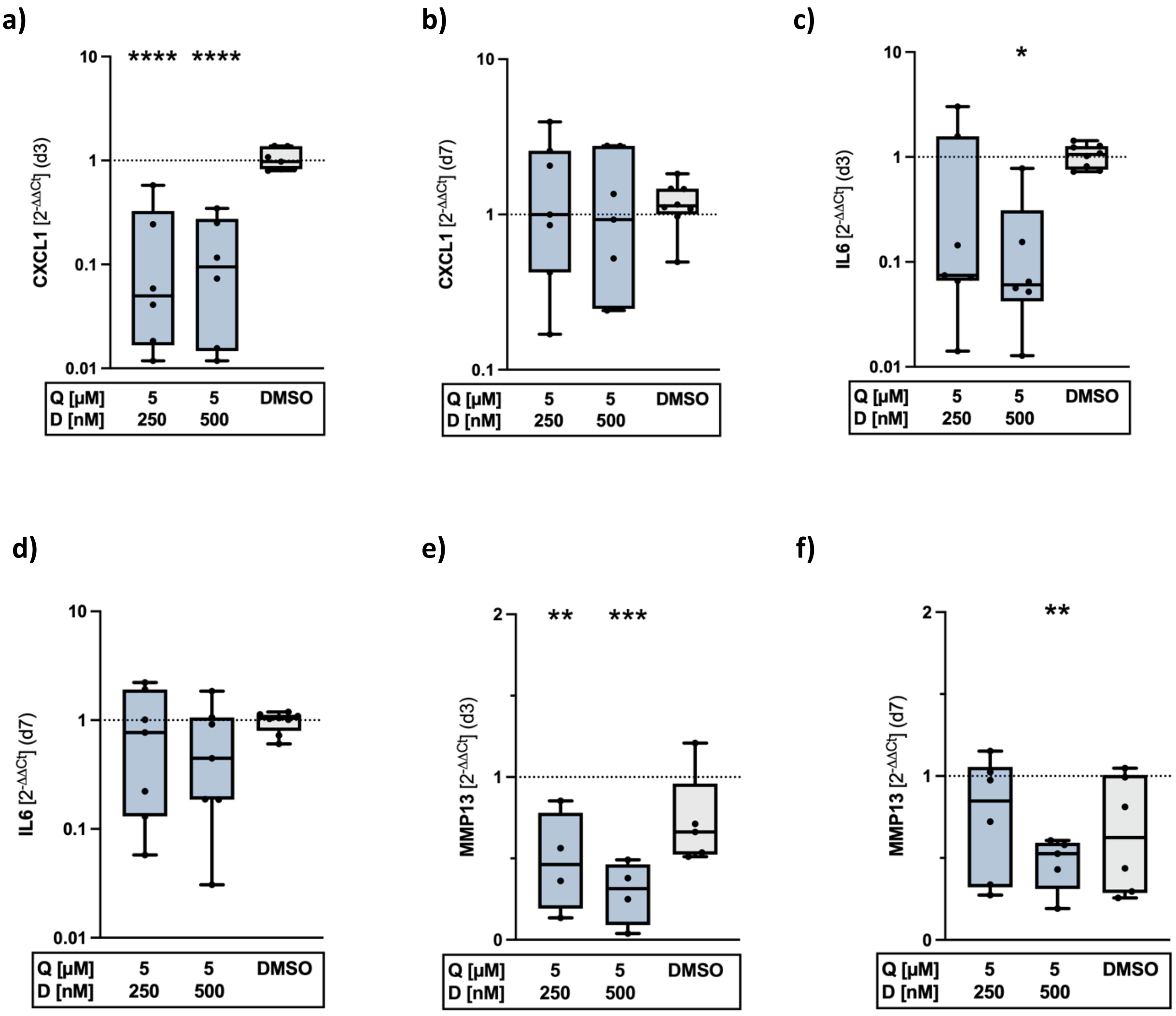

Figure S5: Reduction of SASP factor expression. a-b) Gene expression analysis of CXCL1 (d3+d7) (a) n≥6, b) n≥7), c-d) IL6 (d3+d7) (c)n≥6, d) n≥7), and e-f) MMP13 (d3+d7) (e) n=5, f) n=6). Statistical analysis: one-way ANOVA with Šidák's multiple comparisons test: *p<0.05, **p<0.01, ***p<0.001, ****p<0.0001.
